# Explainable artificial intelligence modeling of internal arc in a medium voltage switchgear based on different CFD simulations

**DOI:** 10.1016/j.heliyon.2024.e29594

**Published:** 2024-04-16

**Authors:** Mahmood Matin, Amir Dehghanian, Mohammad Dastranj, Hossein Darijani

**Affiliations:** a R&D Department, Kerman Tablo Corporation, Kerman, Iran; b Mechanical Engineering Department, Shahid Bahonar University of Kerman, Kerman, Iran; c Department of Mechanical Engineering, Shiraz University of Technology, Shiraz, Iran

**Keywords:** Machine learning, Internal arc of medium voltage switchgear, Computational fluid dynamics, Shapley additive explanation, Extreme gradient boosting

## Abstract

The internal arc represents an unintentional release of electrical energy within the switchgear industry. Manufacturers must address this electro-thermal issue in their switchgears. Over the past decades, various researchers and engineering groups have examined the internal arc pressure rise in switchgears to mitigate damages. The high variability in pressure rise among switchgears due to diverse factors such as design, manufacturing, and electrical parameters results in varying reported pressure increases. This issue motivates the application of artificial intelligence (AI) in interpreting internal arc modeling. The present paper explores the impact of manufacturing parameters such as total duct width (TDW), height (H), and ducts condition (DC), along with environmental parameters like initial pressure (IP) and initial temperature (IT), on the maximum pressure (MP) generated during an internal arc in a medium voltage (MV) switchgear. For this purpose, 54 different computational fluid dynamics (CFD) models were built using the parameters indicated. An extreme gradient boosting (XGBoost) machine learning (ML) model was trained using different CFD models, with MP serving as the target variable for the ML model. The obtained results reveal a variation in the MP of the internal arc under the mentioned parameters, ranging from 17835.45 Pa to 144423.2 Pa. Using SHAP data revealed that IP, TDW, and DC were the most significant factors affecting the pressure increase of the internal arc phenomena.

## Highlights

• Proposed CFD-machine learning model for internal arc pressure estimation.
• The CFD model for switchgear internal arc was validated by an analytical model.
• The dataset was generated by varying manufacturing and environmental parameters.
• Shapely additive explanation method was employed to interpret the XGBoost model.
• Initial pressure, duct width, and condition are key parameters of internal arc.

## 1 Introduction

**Fig. 1:**
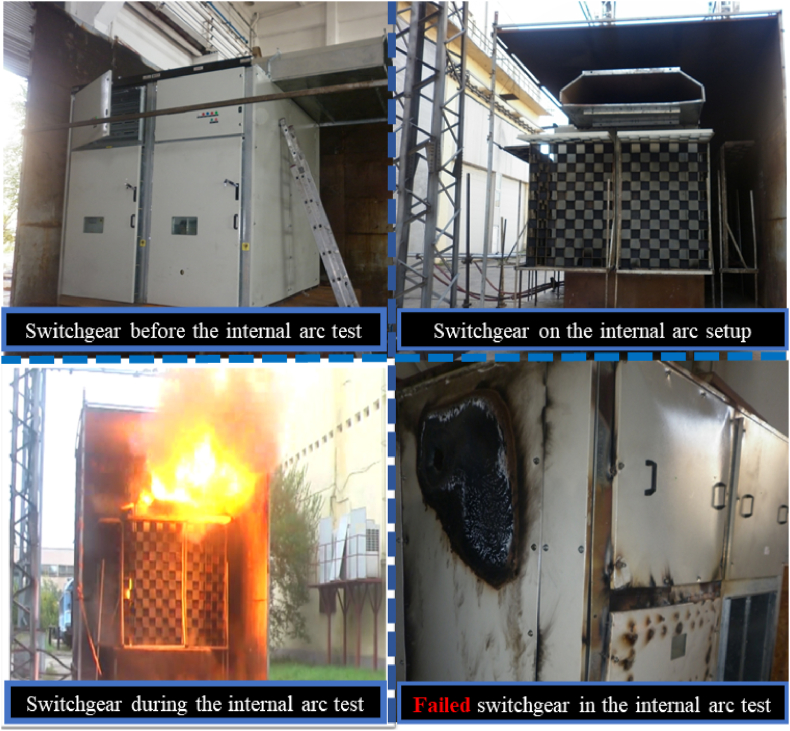
Testing the internal arc of the KMZ_31 switchgear in ICMET high power laboratory, Romania.

An internal arc fault within switchgear is an accidental discharge of electrical energy, leading to short-circuit currents flowing between various phases and to the ground. Furthermore, arcing generates heat within the switchgear enclosure, which leads to an increase in filling gas pressure [1]. An internal arc fault is uncommon, but when it occurs, it can cause substantial damage to both electrical equipment and buildings, potentially endangering humans [2]. The internal arc simulation tool serves as a valuable asset for enhancing design efficiency and safety, particularly in cases where conducting real-world tests is either not feasible or impractical [1]. Fig. 1 illustrates the KMZ_31 switchgear during an internal arc test in a high-power laboratory, where it did not meet the required criteria due to excessive gas pressure.

The design of switchgear panels must prioritize considerations such as the maximum pressure (MP), the rate at which pressure rises, and the spatial distribution of pressure to ensure structural integrity [3]. Additionally, the internal arc pressure rise significantly influences various parameters. Deb et al. [4] conducted a study to examine the fundamental assumptions and design parameters of MV switchgear to investigate internal arc occurrences. They introduced 12 distinct electrical and mechanical parameters for the internal arc investigation. The effect of filling gas in switchgear, as one of the significant parameters in the pressure rise of internal arcs, has been investigated by various researchers [1,5,6]. Reichert and Petchanka [6] address internal arc faults using a 3D CFD model and compare three insulating gases: SF6, air, and CO2. Moreover, they corroborated their conclusions through experimental pressure increases using air-insulated gas as a reference. Furthermore, the examination of different frequencies related to both alternating current and direct current arcing, along with the evaluation of arc energy input [7] and energy absorbers [8], were conducted in the literature. Additionally, geometrical parameters of switchgear have been studied in separate research efforts to explore their impact on the pressure rise during internal arcs, such as exhaust duct area [1] and cover situations [9]. Due to the influence of internal arcs under various conditions, Uzelac et al. [10] created the "Basic Model," an analytical model with considerable limitations for predicting pressure rises in cubic enclosure geometries. They conducted a sensitivity analysis for the main parameters of internal arc phenomena and illustrated the effects of various environmental factors, including IP and IT. Moreover, they introduced a CFD model with a 3D geometry for validation alongside the basic model. The primary limitation of studies investigating internal arcs lies in the high variability and dependency of pressure rise on several variables. These studies often examine variables that are initially unrelated to each other. Therefore, there is no precise comprehension of which parameter, level by level, has a more significant impact on the pressure rise of the internal arc.

Due to the high cost of CFD and its time-consuming nature, especially for transient complex problems, as well as the high variability in some CFD models, AI methods can aid in optimizing variables and understanding the influence of different variables on the outputs [11]. Mohammadi et al. [12] examined how specific design elements of purpose-built baffles impact the performance of a shell and tube heat exchanger through CFD modeling. They compiled a dataset consisting of 27 samples for this analysis. Furthermore, they employed artificial neural networks (ANN) in a sensitivity analysis, which revealed that the design of the baffle cuts had the most significant impact on both heat transfer and pressure drop. CFD modeling of turbulence is challenging, making it difficult to solve problems. Therefore, ML approaches have been added to decrease errors and investigate different scenarios for turbulent modeling based on CFD simulations [11]. In addition to the significant impact of CFD-based turbomachine modeling on various parameters, ML methods have been employed for sensitivity analysis to assess the primary parameters and predict the behavior of these turbomachines based on CFD modeling [13].

The SHAP value method is based on cooperative game theory for interpreting ML models. SHAP values have been utilized to determine the influence of different parameters on the prediction of a target [14]. This method has been used in various fields of engineering, such as the manufacturing of alloys [15], 3D printing [16], and coal mining [17].

Nowadays, switchgear manufacturers have applied AI approaches to improve their traditional manufacturing, monitoring, and maintenance processes. Moon et al. [18] proposed an ML-based method for making remanufacturing decisions for gas-insulated switchgears. Their primary focus was on extending product lifetimes and reducing resource wastage. Additionally, Alsumaidaee et al. [19] conducted airborne ultrasonic testing, a non-destructive testing technique, and recorded audio data during their tests. They utilized deep learning methods to identify and monitor arc faults in switchgears, ultimately enhancing the safety and reliability of power systems. Hoffmann et al. [20] conducted a comprehensive review of the current state-of-the-art in all aspects of condition monitoring for MV switchgear. Furthermore, they presented an approach for developing a predictive maintenance system based on novel sensors and ML techniques. From an alternative perspective, in the manufacturing of MV switchgear, each manufacturer records a pressure rise based on their specific geometry. Rochette et al. [21] exemplified the utility of this approach by comparing simulation and experimental outcomes for internal arc issues, demonstrating a pressure rise of $1.8\times 10^{5}$ Pa with their specific geometry. Similarly, Reichert and Petchanka [6] illustrated a Pressure-Time plot for an air-filled gas switchgear, documenting a pressure increase of $4\times 10^{5}$ Pa through both experimental and simulation results. Additionally, Dullni et al. [1] investigated a pressure rise of $2\times 10^{5}$ Pa within their geometry. Therefore, the motivation for incorporating AI methods in the switchgear manufacturing industry could help understand internal arcs within switchgear. This involves determining the optimum geometry to prevent pressure rise during internal arcs.

The present paper innovatively investigates the impact of various environmental and non-standard manufacturing parameters on the MP in internal arc phenomena within MV switchgears. This investigation is based on a CFD dataset, with SHAP values providing practical guidance for switchgear manufacturers. Additionally, it highlights the effectiveness of ML approaches in predicting the MP in internal arc occurrences within switchgears.

## 2 Research Methods

Basic modeling and CFD modeling are two typical methods for analyzing internal arc problems, and they will be briefly presented below. Furthermore, this section will explain the CFD dataset settings and the ML algorithms used in this paper.

### 2.1 Basic method

**Fig. 2:**
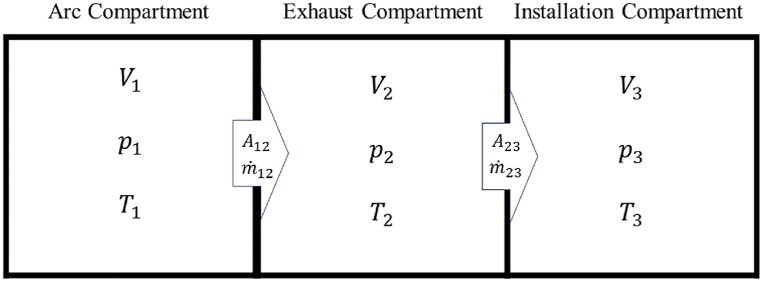
Arrangement of compartments and quantities for internal arc problem [10].

This method is a simple approach for obtaining the variation of pressure and temperature inside the volume over time, and the dependency of these quantities on the geometry is not considered. Fig. 2 illustrates the arc and exhaust compartments and the installation room or environment [10].

Internal arc input energy of $Q_{1}$ ignites to the $V_{1}$ compartment. A relief opening with cross-section $A_{12}$ connects the arc compartment to the exhaust compartment $V_{2}$. When the pressure in the first compartment $p_{1}$, reaches the response pressure, the relief opens and the gas flows into the $V_{2}$ compartment. Then the gas flows to the installation room or environment through the opening area $A_{23}$.

Arc energy entering the $V_{1}$ compartment can be considered as part of electrical energy at the internal arc phenomenon [10].

(1) $$Q_{1}=k_{p}W_{el}$$

$k_{p}$ is the thermal transfer coefficient and can be kept constant [10].

The temperature change of gas inside the arc and exhaust compartments after the time span Δt can be obtained by the following equations [10].

(2a) $$\Delta T_{1}=\frac{\Delta Q_{1}-\Delta m_{12}\left(c_{p1}-c_{v1}\right)T_{1}}{m_{1}c_{v1}}$$

(2b) $$\Delta T_{2}=\frac{\Delta m_{12}\left(c_{p1}T_{1}-c_{v2}T_{2}\right)-\Delta m_{23}\left(c_{p2}-c_{v2}\right)T_{2}}{m_{2}c_{v2}}$$

In the above equations $\Delta m_{12}$, $\Delta m_{23}$, $c_{p}$, $c_{v}$, $m_{1}\text{,}$ and $m_{2}$ are the amount of mass leaving compartment $V_{1}$, the amount of mass leaving compartment $V_{2}$, specific heat capacity at constant pressure, specific heat at constant volume, mass of the gas inside the compartment $V_{1}$ and mass of the gas inside the compartment $V_{2}$, respectively.

The pressure of the gas inside the enclosures is obtained with the aim of ideal gas relation as follows:

(3a) $$P_{1}=\frac{\left(\kappa_{1}-1\right)}{V_{1}}m_{1}c_{v1}T_{1}$$

(3b) $$P_{2}=\frac{\left(\kappa_{2}-1\right)}{V_{2}}m_{2}c_{v2}T_{2}$$

κ is the ratio of specific heat capacity at constant pressure to specific heat capacity at constant volume.

It is common to use the following equation for obtaining the arc input power [10].

(4) $$\dot{Q}=\frac{2\sqrt{2}}{\pi }N_{phase}k_{p}U_{arc}I_{rms}$$

$U_{arc}$, $I_{rms}\text{,}$ and $N_{phase}$ are arc voltage between phases, effective short circuit current, and number of phases, respectively.

### 2.2 CFD modeling

This section explains the general equation for internal arc phenomena CFD modeling. Subsequently, the properties of the solved CFD models in COMSOL Multiphysics software will be detailed.

The CFD model presented in this work is based on conservation laws including mass, momentum, and energy [22]. For incompressible flow inside the enclosure, conservation laws are as follows [22]:

(5) $$\frac{D\rho }{Dt}+\rho \nabla .\vec{V}=0$$

(6a) $$\rho \frac{Du}{Dt}=-\frac{\partial \sigma_{x}}{\partial x}+\frac{\partial \tau_{xy}}{\partial y}+\frac{\partial \tau_{xz}}{\partial z}+F_{x}$$

(6b) $$\rho \frac{Dv}{Dt}=-\frac{\partial \sigma_{y}}{\partial y}+\frac{\partial \tau_{yx}}{\partial x}+\frac{\partial \tau_{yz}}{\partial z}+F_{y}$$

(6c) $$\rho \frac{Dw}{Dt}=-\frac{\partial \sigma_{z}}{\partial z}+\frac{\partial \tau_{zx}}{\partial x}+\frac{\partial \tau_{zy}}{\partial y}+F_{z}$$

(7) $$\rho \frac{Dh}{Dt}=\nabla .\left(k\nabla T\right)+\dot{Q}+\frac{Dp}{Dt}+\mu \varphi$$

Where ρ is the density of gas, V is the vector of velocity that consists *u*, *v,* and *w* along *x*, *y* and *z* directions, respectively, σ and τ are normal and shearing stresses that act on element, respectively, *F* is the body force, *h* is enthalpy of gas, *k* is the thermal conductivity, μ is the dynamic viscosity and μφ is the viscous dissipation term.

The domain of solving will be divided into small cells and then coupled mass, momentum, and energy equations will be solved [22]. To solve the above equations, the ideal gas equation which relates the density of gas with its temperature and pressure should be involved [8,23].

#### 2.2.1 CFD modeling for the 3D validation problem

**Fig. 3:**
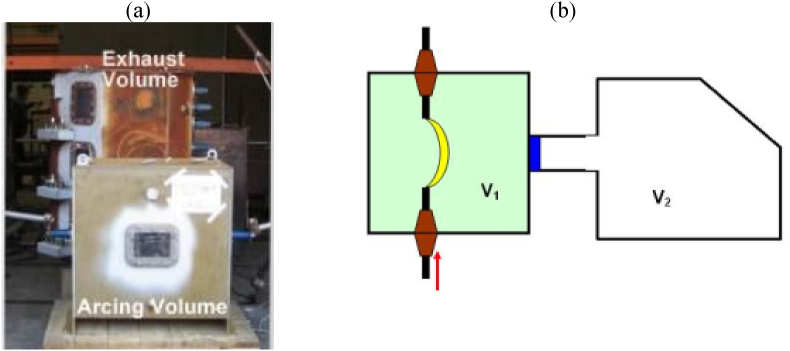
(a) and (b) Views of geometries considered for validation [10].

For validation of the CFD modeling methodology used in this paper, the geometry shown in Fig. 3 is employed. This geometry corresponds to experimental data on internal arc pressure rise as documented in the literature [10].

Fig. 3 illustrates the geometry of two cubic boxes. One of them, known as the 'arcing volume' (V1), is where the arc occurred. The other one is 'exhaust volume' (V2), which refers to the transfer of high-pressure and high-temperature gas from the arcing gas. Between these cubic volumes is a pressure relief valve, which is not opened until the pressure inside the arcing volume reaches the relief opening's response pressure. In the present paper, the validation of the CFD model involves comparing the pressure rise in the V1 compartment to the experimental pressure rise and basic modeling method. Furthermore, Table 1 includes details about the physical and geometrical factors.

**Table 1:** Parameters and initial values for validation problem.

Name of parameter or initial value	Value	Unit
Volume of arc comp.	0.509	$m^{3}$
Initial Pressure in $V_{1}$	160	kPaabsair
Area of relief opening in $V_{1}$	0.00456	$m^{2}$
Response pressure of relief opening	285	kParel
Short-circuit current	14.5	kArms
Number of phases	1	
Averaged phase-to ground voltage	424	V
$k_{p}$ coefficient	0.55	

**Fig. 4:**
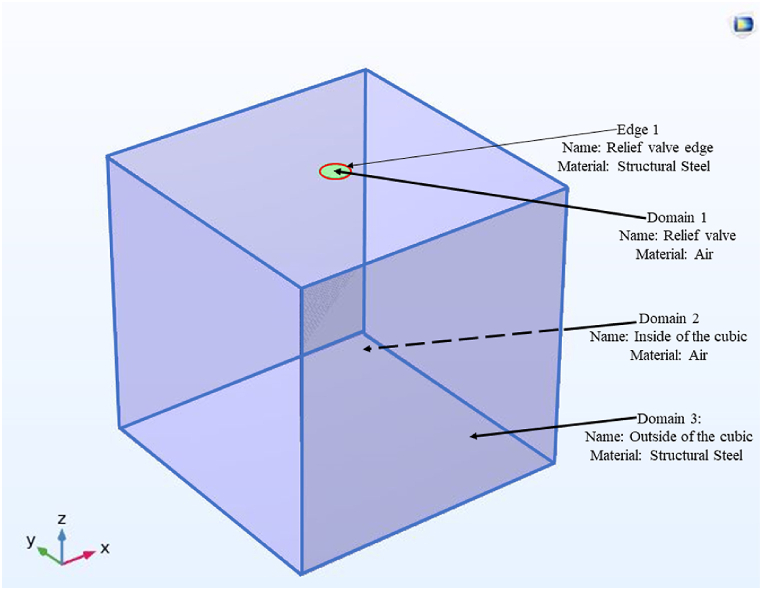
CFD domain and boundaries of the validation problem.

The CFD modeling of the validation problem comprises two steps. The first step involves the scenario where the relief valve remains closed until the air pressure reaches the response pressure for relief valve opening, as specified in Table 1. The second step occurs when the pressure rise surpasses the response pressure for the relief valve opening. Moreover, it is essential to note that all boundaries are considered wall boundary conditions while investigating the pressure rise during the closed relief valve step. Furthermore, the transient study initiates from 0 s and continues until the pressure surpasses the response pressure for the relief valve opening. Fig. 4 depicts the domain and geometry of the validation problem. Table 2 shows the physical properties of the validation problem in COMSOL Multiphysics.

**Table 2:** Physical properties of COMSOL Multiphysics software during closed relief valve configuration.

Physics	Sub-Physics	Notable Sub-Physics Properties
Fluid Flow	Fluid Properties 1	Domain: 1,2
		Constitutive relation: Newtonian
		Dynamic viscosity: From material
	Initial Values 1	Domain: 1,2
		Velocity field: $u_{x}=0\frac{m}{s},u_{y}=0\frac{m}{s},u_{z}=0\frac{m}{s}$
		Pressure:160000 Pa
	Wall 1	Domain: 3, Edge 1
		Wall condition: No slip
Heat Transfer in Fluids	Fluid 1	Thermal conductivity: From material
		Fluid type: From material
	Initial Values 1	Domain:1,2
		Temperature: 293.15 K
	Thermal Insulation 1	Domain: 3, Edge 1
		Show equation assuming: **study 1**^a^
	Heat Source 1	Domain: 1,2
		Show equation assuming: study 1
		Heat source: Q: User defined Q $=Q_{m}$^b^
Multiphysics	Nonisothermal Flow 1	First Physic: Fluid flow
		Second Physic: Heat Transfer in Fluids
		Specify density: From heat transfer interface
		Specify reference temperature: From heat transfer interface
		Flow heating: Include viscous dissipation

a Study1: The transient study.

b $Q_{m}$: General heat source with a unit of $\frac{W}{m^{3}}$.

In Table 2, Table 3, $Q_{m}$ represents the arc power obtained from Equation (4), where this value is divided by the volume of the compartment. This particular value stands out as the most significant initial condition for the CFD modeling. It is advisable to specify the variables of Equation (4) in the parameters section of COMSOL to acquire this value. Table 1 displays all the variables of Equation (4) for the validation geometry.

**Table 3:** Physical properties of COMSOL Multiphysics software during opened relief valve configuration.

Physics	Sub-Physics	Notable Sub-Physics Properties
Fluid Flow	Fluid Properties 1	Domain: 1,2
		Constitutive relation: Newtonian
		Dynamic viscosity: From material
	Wall 1	Domain: 3
		Wall condition: No slip
	Outlet1	Domain: Edge 1
		Average pressure: 285000Pa
Heat Transfer in Fluids	Fluid 1	Thermal conductivity: From material
		Fluid type: From material
	Thermal Insulation 1	Domain: 3
		Show equation assuming: **study 1**^a^
	Heat Source 1	Domain: 1,2
		Show equation assuming: study 1
		Heat source: Q_0_: User defined Q_0_$=Q_{m}$^b^
	Outflow1	Domain: Edge 1
		Show equation assuming: **study 1**
Multiphysics	Nonisothermal Flow 1	First Physic: Fluid flow
		Second Physic: Heat Transfer in Fluids
		Specify density: From heat transfer interface
		Specify reference temperature: From heat transfer interface
		Flow heating: Include viscous dissipation

a Study1: The transient study.

b Q_m_: General heat source with a unit of $\frac{W}{m^{3}}$.

In the second step of obtaining the pressure rise in the validation problem, once reaching the response pressure of the relief opening (285,000 Pa) at 0.093 s, the transient CFD model should be solved from 0.093 s onwards. Moreover, this involves specifying the outflow properties and adjusting the initial pressure to the response pressure of the relief opening. Table 3 presents the physical properties for the second step of the problem in COMSOL Multiphysics.

#### 2.2.2 CFD modeling of KMZ_31 MV switchgear

**Fig. 5:**
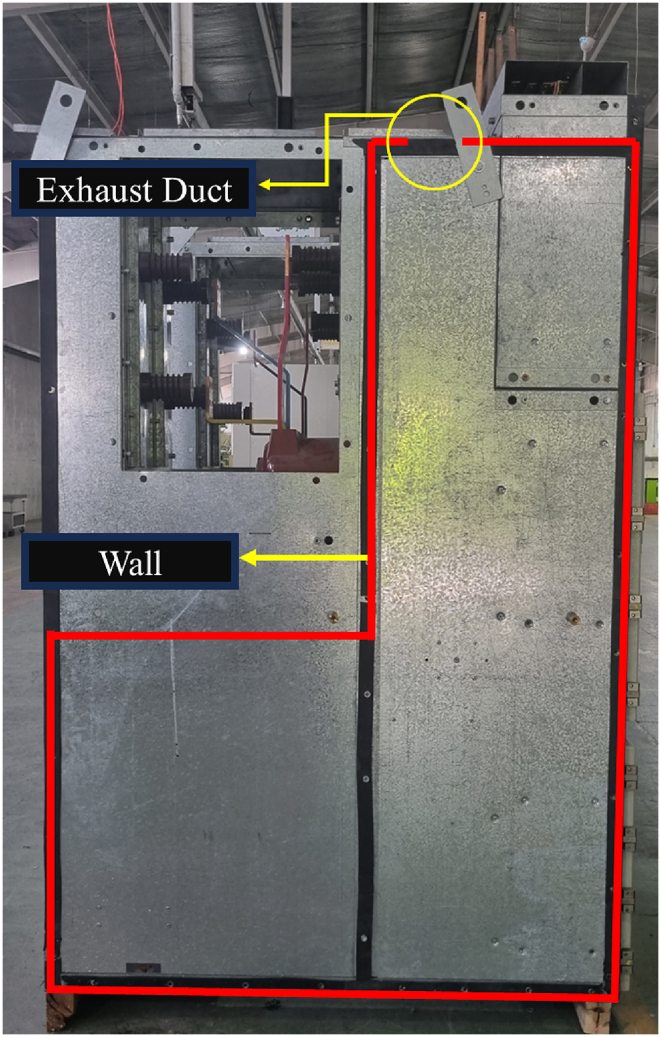
The KMZ_31 switchgear, which served as the case study for CFD modeling and the generation of the CFD dataset, is discussed in this paper.

The previous section explained the CFD modeling methodology for the internal arc. This section delves into an examination of the geometry of the KMZ_31 MV switchgear, with Fig. 5 illustrating the considered geometry for this study.

The width adjustment of this switchgear type, about standard circuit breakers and switchgear manufacturers, poses challenges for modification. Moreover, the computational intricacy inherent in 3D CFD modeling for internal arcs within switchgear, as discussed in the preceding section with simple geometry, imposes substantial demands on system memory resources. Additionally, this is particularly true when solving over 50 instances of the CFD model with varying geometric parameters. As a result, the current study explores a 2D geometry for the case study.

Generally, the internal arc test for MV switchgear is organized by IEC 62271-200 requirements. Table 4 illustrates the main factors used to obtain the arc power of the KMZ_31 switchgear in Equation (4). These parameters represent the highest level of internal arc power simulated in the internal arc test based on IEC 62271-200.

**Table 4:** Parameters and initial values for KMZ_31 problem.

Name of parameter or initial value	Value	Unit
Short-circuit current	31.5	kArms
Number of phases	3	
Arc voltage	380	V
Rated duration of internal fault current	0.25	s
$k_{p}$ coefficient	0.55	

**Fig. 6:**
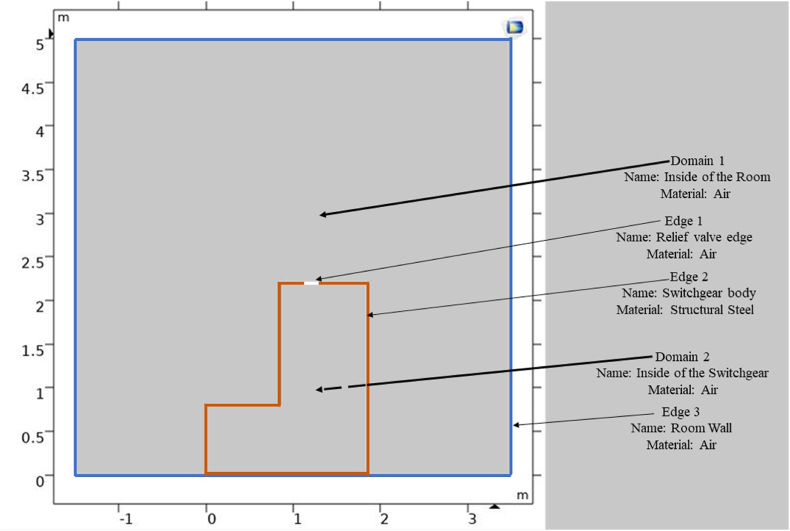
CFD domain and boundaries of the switchgear problem with one exhaust duct.

The CFD model of the KMZ_31 switchgear features an open relief pressure, and all exhaust ducts are in an open state. The switchgear is positioned in an installation room with dimensions of 5 m × 5 m, making the geometry and problems closely resemble reality. Fig. 6 illustrates the domain and geometry of the switchgear problem in COMSOL Multiphysics with one exhaust duct.

The CFD modeling of internal arc switchgear necessitates the incorporation of a transient solver. The transient evolution of this phenomenon spans from 0 to 0.25, as outlined in Table 4. Furthermore, an analysis will demonstrate the dependency of the transient solution on the MP about time steps.

The mesh type of this model was physics-controlled mesh, and the sensitivity analysis of mesh parameters will be investigated in the following section of the paper. Moreover, in the mesh sensitivity analysis, four distinct factors will be elucidated in the mesh sensitivity analysis: maximum element size, minimum element size, element growth rate, and curvature factor. The curvature factor is a geometry-dependent parameter that influences mesh reinforcement. A higher curvature factor results in a more refined mesh in high curvature zones, whereas a lower curvature factor allows for a coarser mesh. The growth rate dictates the speed at which the size of elements increases from one mesh layer to the next. A smaller growth rate results in a more gradual transition between element sizes. Table 5 summarizes the physical characteristics required to solve the CFD model of the geometry shown in Fig. 6. The heat rate, denoted as Q in this table, is the same as the arc power in Equation (4).

**Table 5:** Physical properties of COMSOL Multiphysics software for the KMZ_31 switchgear problem.

Physics	Sub-Physics	Notable Sub-Physics Properties
Fluid Flow	Fluid Properties 1	Domain: 1,2
		Constitutive relation: Newtonian
		Dynamic viscosity: From material
	Initial Values 1	Domain: 1,2
		Pressure:0 Pa
	Wall 1	Domain: Edge 2, Edge 3
		Wall condition: No slip
Heat Transfer in Fluids	Fluid 1	Thermal conductivity: From material
		Fluid type: From material
	Initial Values 1	Domain: 1,2
		Temperature: 293.15 K
	Thermal Insulation 1	Domain: Edge 2, Edge 3
		Show equation assuming: **study 1**
	Heat Source 1	Domain: 2
		Contributed with: Initial Values 1, Fluid 1
		Show equation assuming: **study 1**^a^
		Heat source: Heat rate $P_{0}=$**Q**^b^
Multiphysics	Nonisothermal Flow 1	First Physic: Fluid flow
		Second Physic: Heat Transfer in Fluids
		Specify density: From heat transfer interface
		Specify reference temperature: From heat transfer interface
		Flow heating: Include viscous dissipation

a Study1: The transient study.

b Q: Heat rate with a unit of W.

### 2.3 CFD dataset

**Fig. 7:**
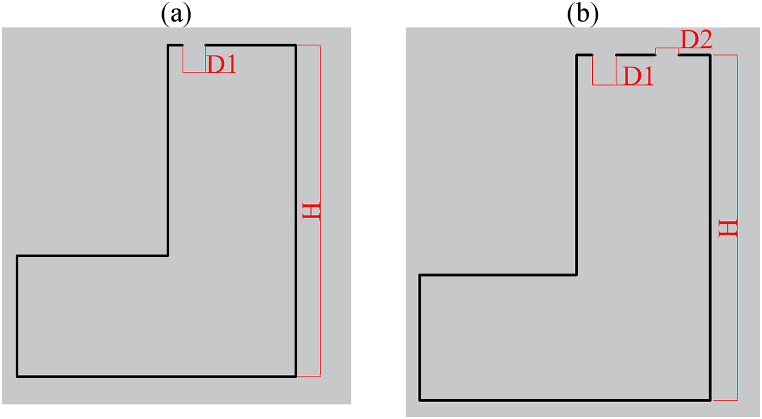
H and TDW Definitions for Single Duct and Two Duct Switchgear a) In single duct switchgear: TDW = D1 b) In two-duct switchgear: TDW = D1 + D2.

**Fig. 8:**
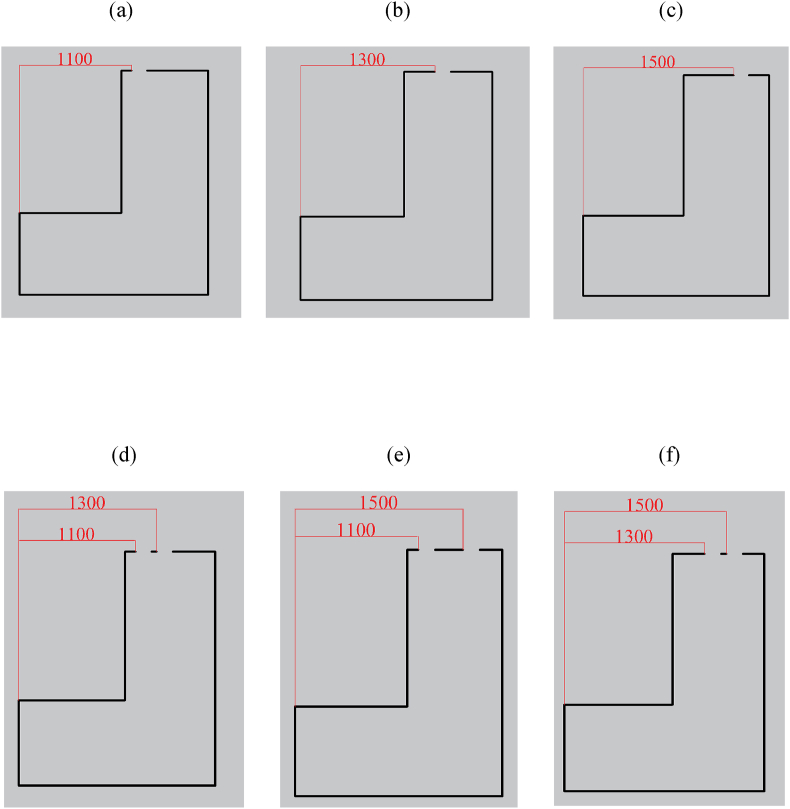
The DC definition of six different geometric conditions represented as follows (units are in mm): a) Condition 1, b) Condition 2, c) Condition 3, d) Condition 4, e) Condition 5, f) Condition 6.

A CFD dataset has been studied for the MP during internal arc phenomena. This dataset contains various parameters that depend on both environmental and geometric conditions. Users can control the environmental variables, including IT and IP, which can be adjusted at the installation location. Furthermore, manufacturers can change certain non-standard geometric variables inside these panels, especially in the context of switchgear, allowing for modifications as needed. Therefore, these non-standard geometric parameters are illustrated in Fig. 7, Fig. 8. Fig. 7 illustrates the concept of H and TDW as two non-standard design factors in switchgear. Additionally, TDW is equal to D1 when the panel has one exhausting duct, and TDW is equal to the sum of D1 and D2 when the panel has two exhausting ducts. Fig. 8 displays six alternative situations involving the presence of one or two ducts and their placements from left to right. These six diverse representations have been categorized as categorical variables in the dataset, referred to as 'Ducts Condition' (DC).

Overall, the CFD dataset comprises two environmental variables (IP and IT) and three design variables (TDW, DC, and H), with MP indicating the target value for each sample.

The explanation details the criteria for picking variations in the CFD dataset for design variables. The IP varies from −40 kPa to +40 kPa. This range allows heating, ventilation, and air conditioning (HVAC) experts to control the environmental conditions of the installation room. IT ranges from 253 K to 318 K, providing a suitable range for air conditioning in the installation room within tolerable limits for humans. H varies between 2 m and 2.6 m, meeting the acceptable criteria for switchgear manufacturers during the design process. The TDW varies between 0.09 m and 0.3 m, which falls within the permitted range set by the manufacturer. However, a thorough investigation is imperative in the stress analysis of the switchgear roof. Moreover, this is essential due to the pressure rise from the internal arc, as an increase in the duct area may actively contribute to a lower-strength roof in the switchgear.

DC serves as a highly dependent variable in the structure of the switchgear, and its specifications are unique to each switchgear based on its initial design.

### 2.4 Machine learning modeling

The Pearson correlation coefficient is used to assess the linear relationship between variables. The following equation demonstrates the Pearson correlation coefficient [24].

(8) $$p\left(a,b\right)=\frac{E\left(a,b\right)}{S_{a}S_{b}}$$

In which variables a and b are different from each other. The Pearson correlation coefficient is denoted as P(a,b), and cross-correlation is represented as E(a,b). The square roots of the variances of a and b are $S_{a}$ and $S_{b}$, respectively. One of the most significant characteristics of the Pearson correlation coefficient is represented in Equation (9). If P(a,b) is near 0, it indicates that a and b are uncorrelated. Conversely, if P(a,b) is close to 1, it shows a strong correlation [24].

(9) $$0\leq p^{2}\left(a,b\right)\leq 1$$

RF is a robust ML algorithm for classification tasks. It is known for its ability to combine multiple weaker classifiers to produce highly accurate results for complex tasks. The RF model is made by building a set of N decision trees. As a result, when making predictions for a given input *x*, the overall prediction is represented as f(x), and $h_{n}\left(x\right)$ the prediction for the *n*-th DT with the same input x. Equation (10) demonstrates this representation [25].

(10) $$f\left(x\right)=\frac{1}{N}\sum_{n=1}^{N}h_{n}\left(x\right)$$

The DT is a commonly used algorithm in ML. It's a hierarchical model that efficiently makes decisions by employing a series of interconnected tests. In each test, it evaluates a numeric feature against a particular threshold value [26]. The objective of a DT is to recursively split the feature space into disjoint regions (leaf nodes) based on the feature values [27].

XGBoost is a supervised ML algorithm that builds upon the concept of gradient-boosted decision trees (GBDT). In the XGBoost model, the estimated output, denoted as $y_{ei}$, is generated by summing up the estimations made by K individual trees, as outlined in Equation (11) [28].

(11) $${y_{e}}_{i}=\sum_{k=1}^{K}f_{k}\left(X_{i}\right)$$

In which, $X_{i}$ denotes the input feature vector, $f_{k}$ represents the estimated scores from the k_th_ tree, and *K* represents the overall count of regression trees. The primary goal of the XGBoost objective function is to reduce the regularized objective function, as outlined in Equation (12) [28].

(12) $$Obj=\sum_{i=1}^{n}l\left(y_{i},y_{ei}\right)+\sum_{j=1}^{K}\Omega \left(f_{k}\right)$$

Where*,* $l\left(y_{i},y_{ei}\right)$ denotes the disparity between the target $y_{i}$ and the estimated $y_{ei}$, ${\Omega (f}_{k})$ represents penalty function and K represents number of trees.

The choice of tree-based algorithms, specifically DT, RF, and XGBoost, stems from their effectiveness in handling mixed categorical and continuous features [29]. DT, known for its simplicity and interpretability, seems to be an appropriate choice for preliminary investigation [29]. RF, as an ensemble of decision trees, not only enhances predictive performance but also mitigates overfitting, striking a balance between complexity and accuracy. XGBoost, a gradient-boosting technique, enhances forecast accuracy by sequentially creating decision trees with a focus on decreasing errors from preceding trees [29]. Moreover, from reviewing the literature, Table 6 compares general information about these three algorithms in ML modeling [29].

**Table 6:** Comparison of ML algorithms used in the present paper based on the literature review.

Feature	XGBoost	DT	RF
Algorithm Type	Boosting	Decision Tree	Ensemble (Bagging)
Handling Missing Values	Yes	No	Yes
Feature Importance	Yes	Yes	Yes
Parallel Processing	Yes	No	Yes
Regularization	Yes	No	Yes (through bagging)
Outlier Sensitivity	Moderate	High	Low (due to averaging)
Hyperparameter Tuning	Required	Limited	Moderate
Use Cases	Various	Simple models, small datasets	Diverse, robust performance

The SHAP values approach is a technique for attributing the importance of each feature to a specific prediction, making it a valuable tool for understanding the interpretation of prediction results. Equation (13) depicts the SHAP values assigned to individual features [30].

(13) $$\varphi_{i}=\sum_{S\subseteq F\backslash \left\{i\right\}}\frac{\left|S\right|!\left(\left|F\right|-\left|S\right|-1\right)!}{\left|F\right|!}\left[f_{S\cup \left\{i\right\}}\left(x_{S\cup \left\{i\right\}}\right)-f_{S}\left(x_{S}\right)\right]$$

Where $\varphi_{i}$ represents SHAP value, F represents the complete model with all its components, and *S* stands for a particular part of the model. S∪{i} is adding a single feature i to S. The model's prediction, taking into account feature i, is denoted as $f_{s\cup \left\{i\right\}}\left(x_{s\cup \left\{i\right\}}\right)\text{.}$ On the other hand, $f_{s}\left(x_{s}\right)$ signifies the model's prediction when i feature was excluded.

In the interpretation of a model using SHAP values, each specified feature in every sample is assigned a SHAP value. Additionally, there is an estimated target value that remains constant across all samples. The SHAP values for each feature in every sample help elucidate the individual contributions of these features to the difference between the model's prediction for a specific sample and the overall expected prediction. This illustrates how changes in features can influence the estimated target value, irrespective of the specific features involved. Equation (14) represents how SHAP values sum to a baseline constant value of the target, consequently estimating the real target value.

(14) $$\hat{y}={\hat{y}}_{0}+\sum_{i=1}^{n}\varphi_{i}$$

Where $\hat{y}$ is the estimated target value, ${\hat{y}}_{0}$ represents the constant estimated target value regardless of the feature influence, and $\sum_{i=1}^{n}\varphi_{i}$ is the sum of the SHAP values in each sample, which is different in each sample.

Hyperparameters play a vital role in governing the performance of models to avoid overfitting in ML methods. In the present paper, hyperparameters were selected based on literature [31], and the grid search cross-validation method was employed to optimize these hyperparameters.

To assess the performance of ML models and compare them to each other, commonly used metrics such as root mean square error (RMSE) and the R^2^, along with graphical representations like scatter band (SB) plots, are typically utilized [15,16]. When *R*^*2*^ is equal to 1, it indicates a perfect model fit, while an *R*^*2*^ lower than 1 suggests errors and lower accuracy in the modeling [15,16]. RMSE measures the average magnitude of errors between predicted values and actual values in a model. Lower RMSE values indicate that the model's predictions are closer to the actual data, while higher RMSE values indicate larger prediction errors [15]. Equation (15) represents the formulation for RMSE.

(15) $$RMSE=\sqrt{\frac{1}{n}\sum_{i=1}^{n}\left(Y_{actual}-Y_{\text{predicted}}\right)}$$

Where *n* represents the number of samples, $Y_{actual}$ denotes the actual values of the target variable (CFD MP in the present work), and $Y_{predicted}$ represents the estimated values of the target variable (estimated MP in this work). In SB plots, predicted values obtained from modeling are compared to existing values, with the horizontal and vertical axes typically scaled using logarithmic transformation. The data points are enclosed between two lines, the slopes of which represent the SB value [15].

The data splitting in the ML models of this study involved various random test sizes of 20 % and random train sizes of 80 %. *R*^*2*^ and *RMSE* were reported by varying the random state during data splitting. Average values were computed over 20 different random states to provide a comprehensive assessment.

Analyzing learning curves is a common method for visualizing and understanding the concepts of overfitting, underfitting, and fitting status about training and testing accuracy. A well-fitted model exhibits elevated accuracy in both training and testing datasets, demonstrating low variance [32].

**Fig. 9:**
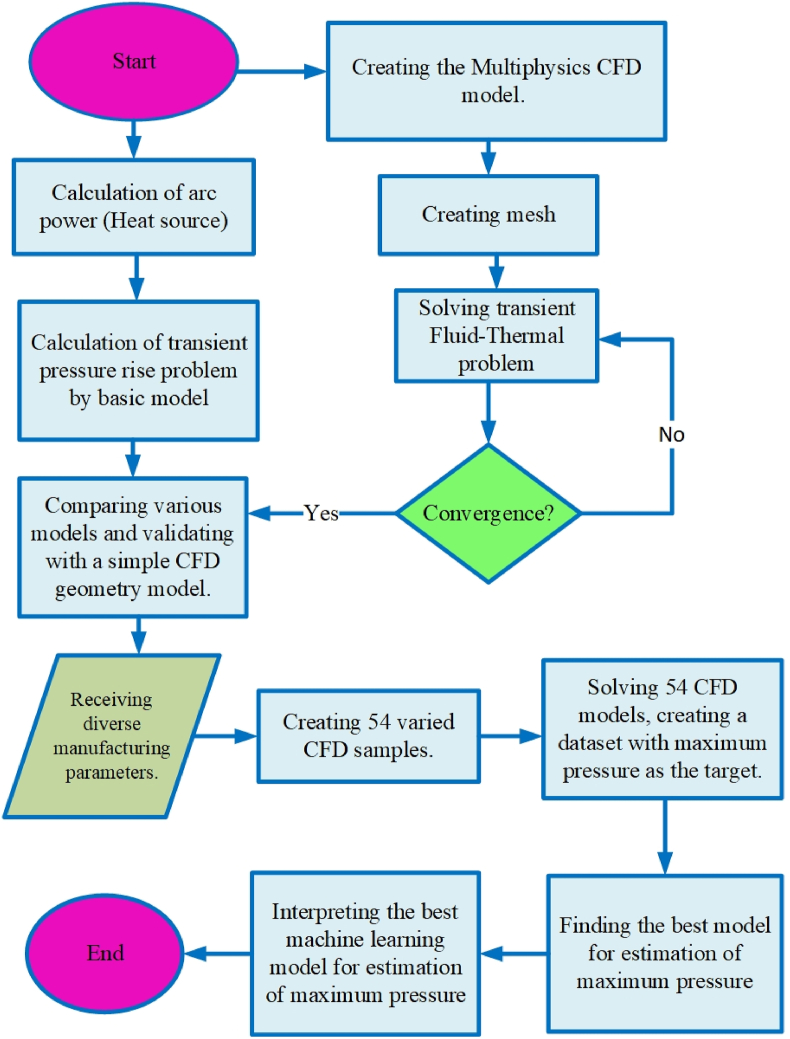
The flowchart of the present modeling.

At the end of the research method section, Fig. 9 illustrates the flowchart of the present work, and the results order is based on this flowchart.

## 3 Results and discussion

The first step in this part is to compare a created CFD model to a basic model with simple geometry and circumstances. Following the validation of the CFD methodology, the CFD modeling is further refined using the KMZ_31 geometry as a case study, and the resulting CFD dataset is reported based on this specific geometry. In the next step, three different ML methods will be compared to estimate the MP based on the CFD dataset. Finally, SHAP values will be utilized to interpret the best ML method among the others.

### 3.1 Validation

**Fig. 10:**
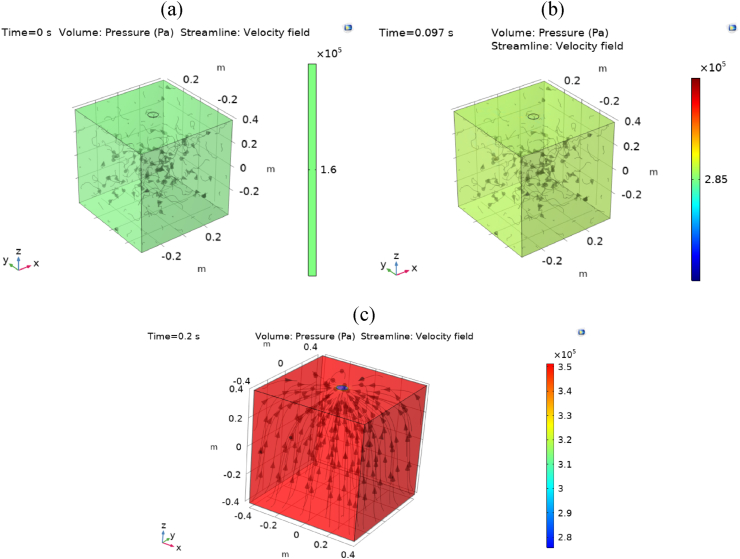
The pressure and streamlines distribution in the validation problem at different times with different relief conditions. (a) Relief is closed (t = 0 s). (b) The relief valve is closed, and pressure applies to the pressure relief opening (t = 0.097s). (c) The pressure was passed from response relief pressure, and the relief is opened (t = 0.2s).

The present paper investigates the internal arc within a simple validation geometry of switchgear using CFD modeling. Fig. 10 illustrates the pressure rise in the arc compartment of the validation geometry over the duration of the internal arc. At t = 0 s, the arc has not initiated, and the IP stands at $1.6\times 10^{5}$ Pa. The opening relief valve remains closed from 0 s to 0.097 s, leading to a pressure increase of $2.85\times 10^{5}$ Pa during this period. This value represents the response pressure associated with the relief valve opening. In Fig. 10(b), the pressure reaches the response pressure of the relief opening, and the streamlines exhibit no specific pattern. After this period, the relief opening activates, initiating a pressure rise that reaches the MP and subsequently diminishes.

In the current paper, an exploration of three distinct methods for determining internal arc pressure rise in enclosures is presented: basic modeling for simple geometries, CFD modeling, and experimental tests. The selection of the geometry depicted in Fig. 4 is driven by the availability of experimental tests designed for this geometry, thereby allowing for the validation of basic modeling in this context.

**Fig. 11:**
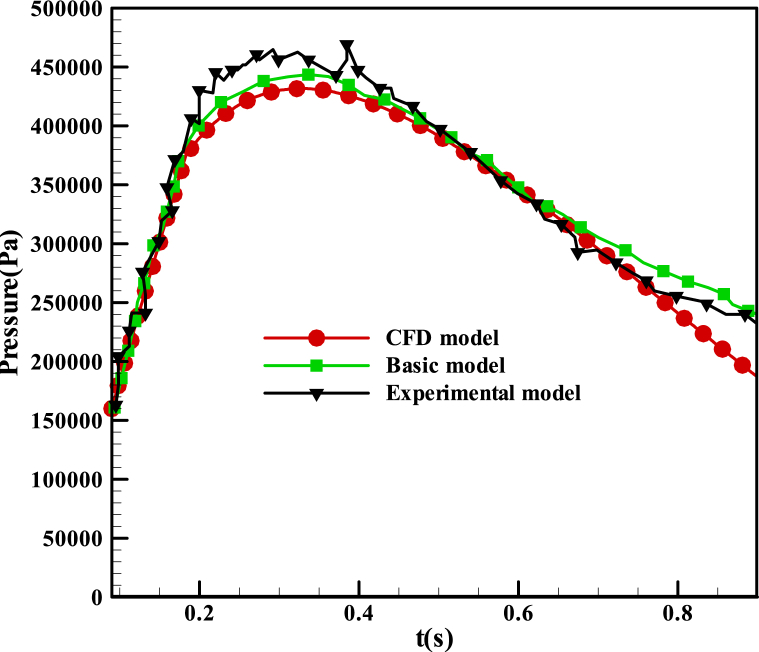
Pressure distribution in the arc compartment of the validation problem.

Fig. 11 compares basic modeling, experimental tests [10], and CFD modeling of the arc compartment for the validation geometry. The previous figure illustrates some of the pressure contours from this CFD modeling. Furthermore, Fig. 11 demonstrates the approval of the CFD modeling of the internal arc following comparison with basic modeling and experimental tests.

### 3.2 CFD dataset

**Fig. 12:**
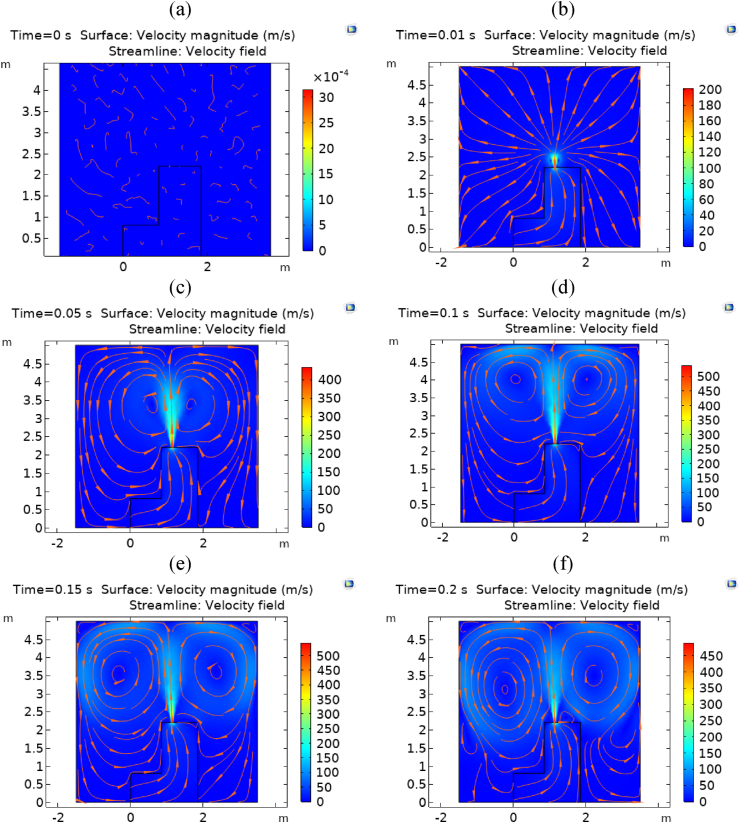
The surface velocity under specified conditions when IP = 0 kPa, IT = 293 K, TDW = 0.09 m, DC = 1, and H = 2.2 m at different times: (a) 0s, (b) 0.01s, (c) 0.05s, (d) 0.1s, (e) 0.15s, and (f) 0.25s.

A dataset was created to explore the effects of several presumed design and environmental switchgear characteristics on the MP of the internal arc occurrence. The basis of the CFD dataset is a CFD model, as illustrated in Fig. 12, representing the surface velocity for a sample problem under specified conditions of its parameters. As can be observed, the complete gas wave is diffused quickly inside the compartment, and the MP within the enclosure is obtained at this time. If the relief aperture for the switchgear was not considered, the pressure wave within the volume would destroy the switchgear's structure, as well as people and everything else in the room. As shown in Fig. 12, the air velocity inside the compartment quickly increases when the internal arc phenomenon starts. However, the gas velocity, particularly near the room walls, starts to decrease as the effects of the arc reach those walls, creating a return wave. It is clear that the relief opening, with its smallest flow cross-section, generates the highest velocity inside the region.

**Fig. 13:**
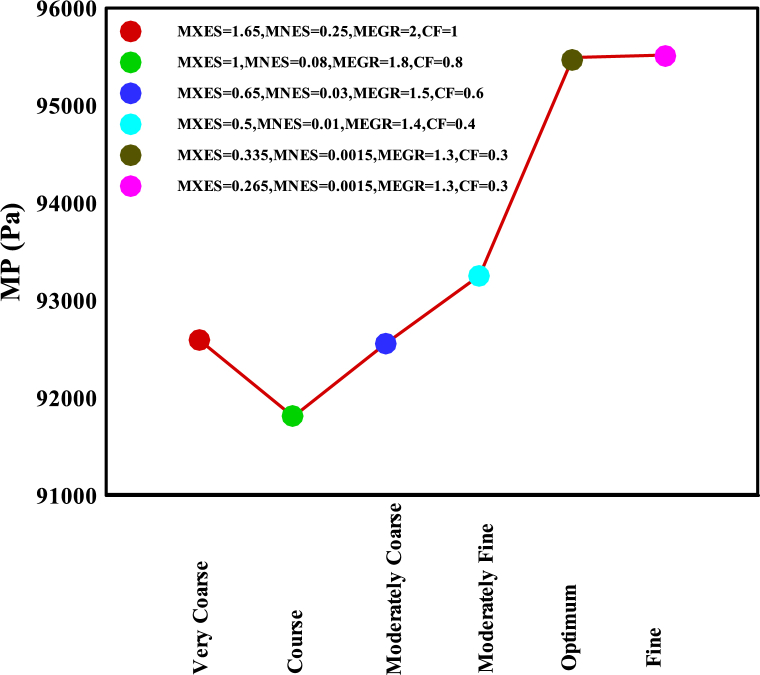
Mesh convergence plot for different adjustable parameters of COMSOL Multiphysics software to obtain the mesh quality for the CFD model in Fig. 12.

Fig. 13 illustrates the mesh convergence sensitivity analysis of adjustable mesh properties using COMSOL Multiphysics software for the CFD modeling of the geometry reported in Fig. 12. In this figure, MXES represents the maximum element size (units in meters), MNES denotes the minimum element size (units in meters), MEGR illustrates the maximum growth rate, and CF demonstrates the curvature factor. The optimal model properties derived from this figure have been utilized to report CFD modeling results. Furthermore, in this model, four mixed elements have been incorporated: triangles, quads, edge elements, and vertex elements.

**Fig. 14:**
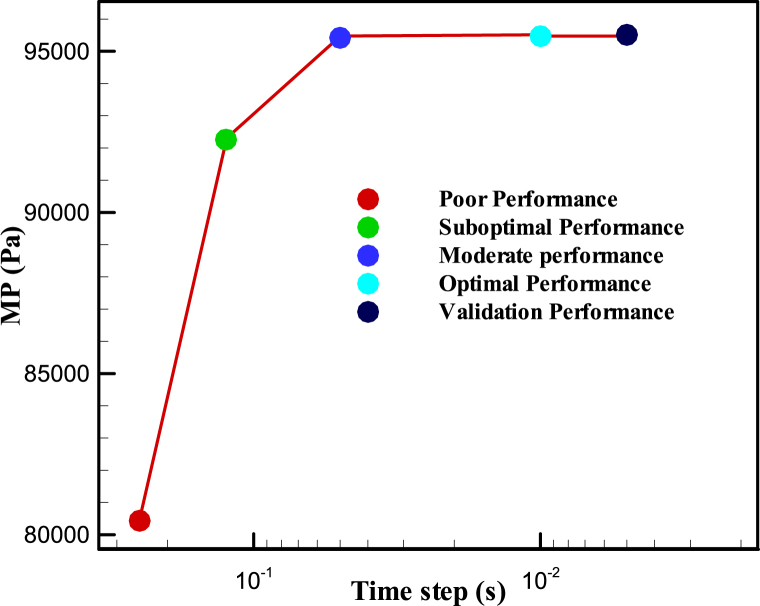
Time step sensitivity analysis to obtain MP in the model shown in Fig. 12.

Fig. 14 illustrates the time-step sensitivity analysis of the transient model reported in Fig. 12. Based on this plot, the optimal value for adjusting the time step to solve the problem was 0.01 s, balancing the cost of time-solving and achieving convergence MP values in a specific region.

**Fig. 15:**
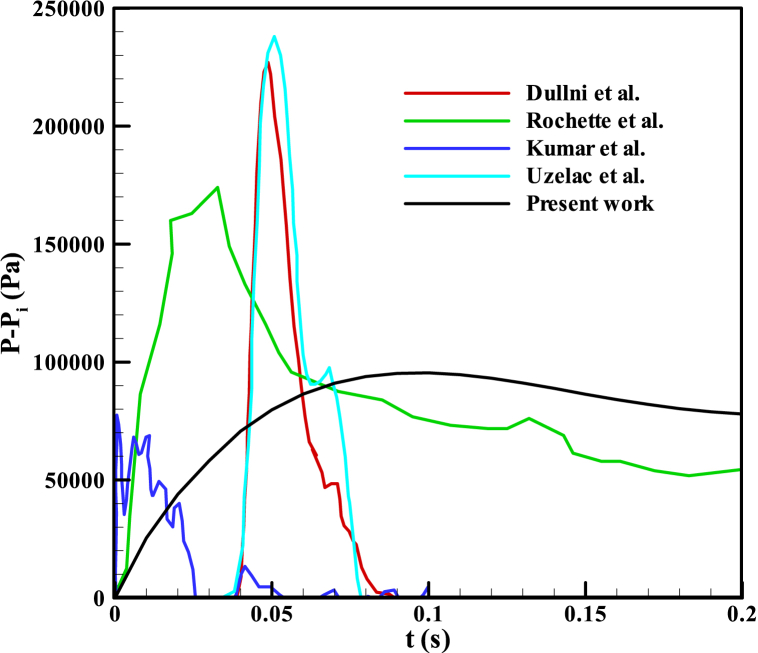
Reported experimental internal arcs pressure rise for MV switchgears in the literature.

After displaying surface velocity in Fig. 12, completing mesh convergence sensitivity analysis in Fig. 13, and performing time step sensitivity analysis in Figs. 14 and 15 compares the pressure rise of the KMZ_31 geometry to various MV switchgears based on literature. Notably, the design parameters of KMZ_31 in these figures (Fig. 12, Fig. 13, Fig. 14, Fig. 15) are the same. Furthermore, Table 7 provides general electrical internal arc information for the switchgears detailed in Fig. 15. As observed in Fig. 15, the pressure rise in switchgears exhibits significant variability and dependency on different conditions, resulting in the absence of a specific MP. As a result, each manufacturer should do optimization based on their design assumptions. This highlights the research gap addressed in the present paper, emphasizing the ongoing need for investigations into switchgear design to accommodate the evolving requirements in progress.

**Table 7:** Internal arc information of MV switchgears represented in Fig. 15.

References	Number of phases	Short-circuit current (kA)	Arc voltage (V)
Dullni et al. [1]	3	35	300
Rochette et al. [21]	3	20	–
Kumar et al. [3]	3	40	–
Uzelac et al. [10]	3	38.8	250
Present Work	3	31.5	380

Table 8 shows the CFD dataset. To calculate the MP for each sample, the parameters specified in the Research Methods section were changed, followed by solving the corresponding CFD problems. Then, the MP was extracted from the pressure and time plot of the critical element of each CFD model. In other words, the findings shown in the last column of each row in Table 8 are obtained by solving the CFD issue using data from other columns. The overall geometry follows the last problem discussed in this section.

**Table 8:** The CFD dataset.

sample	Initial Pressure (*IP*) [kPa]	Initial Temperature (*IT*) [K]	Total Duct Width (*TDW*)---	Ducts Condition (*DC*) [m]	Height (*H*) [m]	Maximum Pressure (*MP*) [Pa]
1	20	253	0.27	4	2.2	53424.11
2	14	307	0.3	6	2.52	42281.41
3	0	293	0.09	1	2.2	95402.81
4	10	262	0.09	1	2	111295.7
5	−10	273	0.09	1	2.1	86676.67
6	40	293	0.228	4	2.2	76702.56
7	−20	278	0.09	1	2.2	74644.88
8	30	283	0.09	1	2.1	126091.5
9	−14	289	0.258	6	2.54	20094.37
10	−30	300	0.09	1	2.3	59460.84
sample	Initial Pressure (*IP*) [kPa]	Initial Temperature (*IT*) [K]	Total Duct Width (*TDW*) ---	Ducts Condition (*DC*) [m]	Height (*H*) [m]	Maximum Pressure (*MP*) [Pa]
11	17	286	0.108	1	2.4	99485.29
12	17	264	0.24	5	2.24	53851.69
13	−40	253	0.108	1	2.2	42979.49
14	26	311	0.108	1	2.5	103632.1
15	−20	293	0.09	2	2.2	72102.66
16	−12	315	0.108	1	2.15	64195.06
17	0	253	0.228	4	2.2	40950.74
18	0	293	0.108	3	2.2	81008.25
19	−40	293	0.108	3	2.2	36406.27
20	20	278	0.108	1	2.2	102494.4
21	−40	318	0.108	1	2.2	32750.39
22	20	293	0.108	3	2.2	100291
23	−20	318	0.108	1	2.2	55047.95
24	−7	305	0.108	1	2.25	71104.21
25	40	253	0.09	2	2.2	144423.2
26	−20	253	0.12	1	2.2	58480.45
27	20	293	0.222	5	2.2	57525.53
28	40	278	0.12	1	2.2	114653
29	4	260	0.12	1	2.35	82861.54
30	0	253	0.24	5	2.2	38777.12
31	−20	318	0.12	1	2.2	48631.77
32	−40	253	0.108	3	2.2	43253.82
33	20	318	0.12	1	2.2	89398.56
34	40	293	0.132	1	2.2	105670.2
35	−25	265	0.132	1	2.45	45081.37
36	40	253	0.108	3	2.2	127982.1
37	−40	253	0.132	1	2.2	30095.93
38	40	293	0.258	6	2.2	70937.87
39	24	270	0.132	1	2.5	99912.38
40	−40	293	0.15	1	2.2	17835.45
41	−16	284	0.15	1	2.55	44463.61
42	−13	269	0.252	5	2.17	22756.55
43	40	293	0.15	1	2.2	97692.07
44	40	253	0.282	5	2.2	69952.41
45	0	253	0.15	1	2.2	65445.17
sample	Initial Pressure (*IP*) [kPa]	Initial Temperature (*IT*) [K]	Total Duct Width (*TDW*) ---	Ducts Condition (*DC*) [m]	Height (*H*) [m]	Maximum Pressure (*MP*) [Pa]
46	−40	253	0.15	1	2.2	22990.83
47	0	293	0.24	4	2.2	35342.6
48	40	253	0.15	1	2.2	103461.9
49	−18	257	0.252	4	2.05	18711.71
50	−40	278	0.15	1	2.2	19621.2
51	−13	297	0.24	6	2.42	22746.27
52	−12	309	0.15	1	2.6	45902.3
53	40	278	0.15	1	2.2	99732.9
54	0	293	0.09	2	2.2	95668.31

### 3.3 Machine learning modeling

**Fig. 16:**
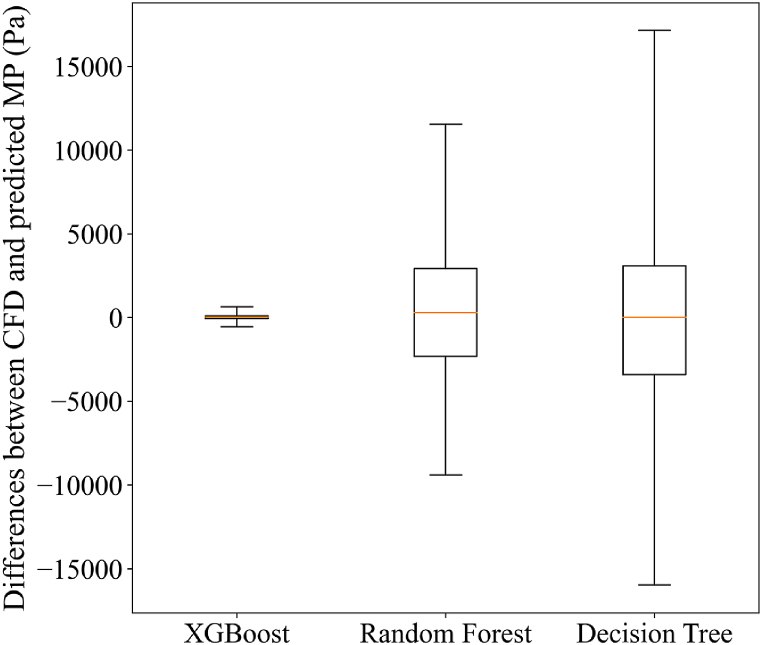
Comparison of the subtraction between actual and predicted MP values when all samples were trained.

Table 9 presents various supervised ML methods for forecasting the MP in internal arc phenomena using the CFD dataset. The metrics are reported as means for both training and testing values after adjusting the random state across the dataset 20 times. Additionally, the hyperparameters for these three different algorithms are reported in this table. Finally, in the last column of this table, the SB values are reported when all 54 data points were trained based on the mentioned hyperparameters. In this table, XGBoost exhibits higher accuracy than RF, consistent with findings in other research studies. Those studies predicted continuous targets using different datasets that included mixed categorical and continuous features [[15], [16], [17],^28^]. Moreover, XGBoost has demonstrated superior performance compared to DT, averaging across more than 10 diverse datasets [33]. Furthermore, Fig. 16 displays a box plot comparing the mentioned ML approaches. This plot illustrates the subtraction between the calculated MP from CFD modeling and the predicted MP when all of the data were trained based on the mentioned hyperparameters in Table 9. Fig. 16 demonstrates that XGBoost had the lowest subtraction between the CFD value of MP and the predicted value of MP, with an error extremely close to zero. Furthermore, based on the physics of pressure, this finding indicates highly accurate modeling with low variance for estimating the MP of the internal arc. Chelgani et al. [28] demonstrated, through a shared box plot, that XGBoost exhibited the lowest error compared to support vector machines and random forests. Nevertheless, in their investigation, the errors of each algorithm clustered closely together. In contrast, the present study reveals that the error plot for XGBoost approaches zero significantly, emphasizing its superior performance compared to the other research.

**Table 9:** Accuracy and hyperparameters of ML-based models for estimating MP.

Model	Hyperparameters	Mean R^2^ (%)		Mean RMSE		SB
		Training	Testing	Training	Testing	
**XGBoost**	n_estimators = 150	**99.99**	**95.49**	**177.04**	**6625.55**	±1.1
	max_depth = 3					
	learning_rate = 0.2					
	subsample = 0.8					
	reg_lambda = 1					
	colsample_bytree = 0.8					
RF	max_depth = 7	98.02	82.83	4873.50	13625.26	±1.2
	'max_features = 'log2					
	min_samples_leaf = 1					
DT	min_samples_split = 2	97.59	81.11	5356.73	13918.80	±1.25
	= 7 max_depth					
	min_samples_leaf = 2					

Note: The bold value means the superior achievement.

**Fig. 17:**
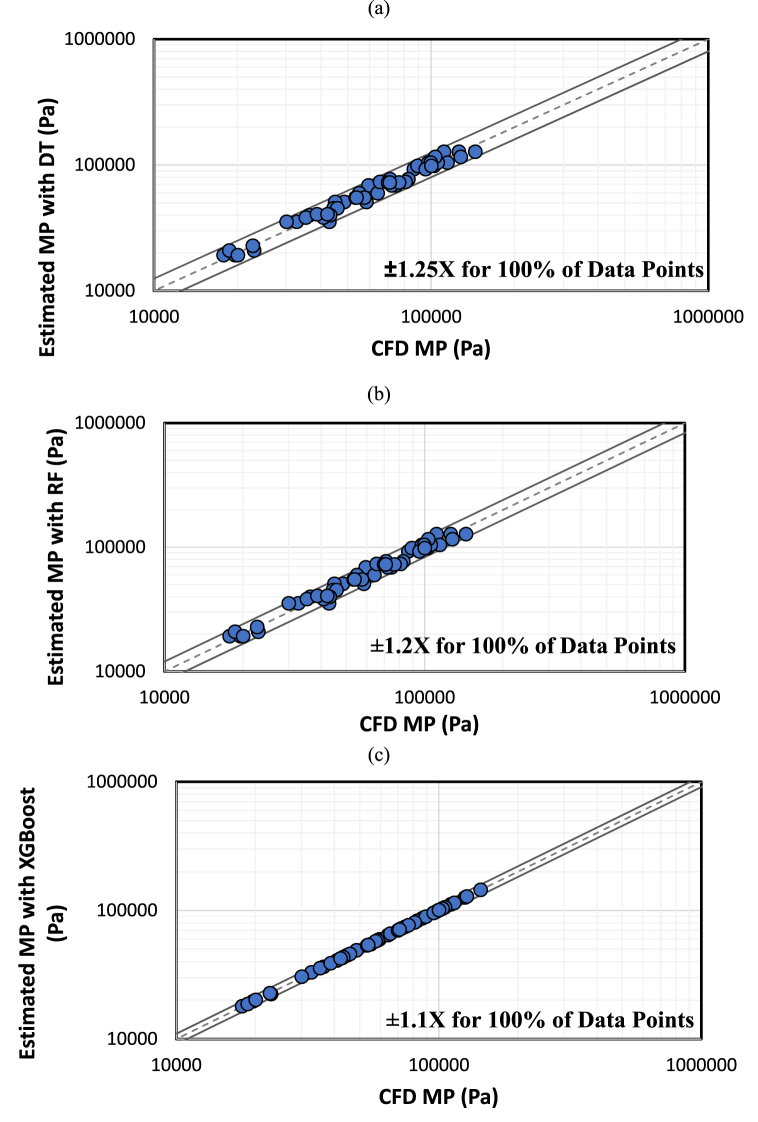
SB for assessing the performance of different ML-based models when all the data were trained based on the mentioned hyperparameters: a) DT, b) RF, c) XGBoost.

Fig. 17 displays the SB plots for all 54 data when they were trained based on the mentioned hyperparameters in Table 9. This figure illustrates that XGBoost had an SB value of ±1.1, RF had an SB value of ±1.2, and DT had an SB value of ±1.25, showing the high performance of XGBoost compared to the others. To compare these results with other research, Matin and Azadi [15] reported the SB to demonstrate the accuracy of their XGBoost model based on an experimental dataset, with a range of approximately ±1.2 for estimating a continuous target. Moreover, XGBoost exhibited a lower SB value compared to RF and support vector machines [16]. This suggests that XGBoost could function as a cost-effective industrial alternative, requiring less financial investment, time, and technical expertise compared to addressing high-variable Multiphysics CFD problems. Therefore, after achieving the best modeling of MP in this work, the ML interpretation has been reported based on XGBoost modeling in the next part of the results and discussion.

**Fig. 18:**
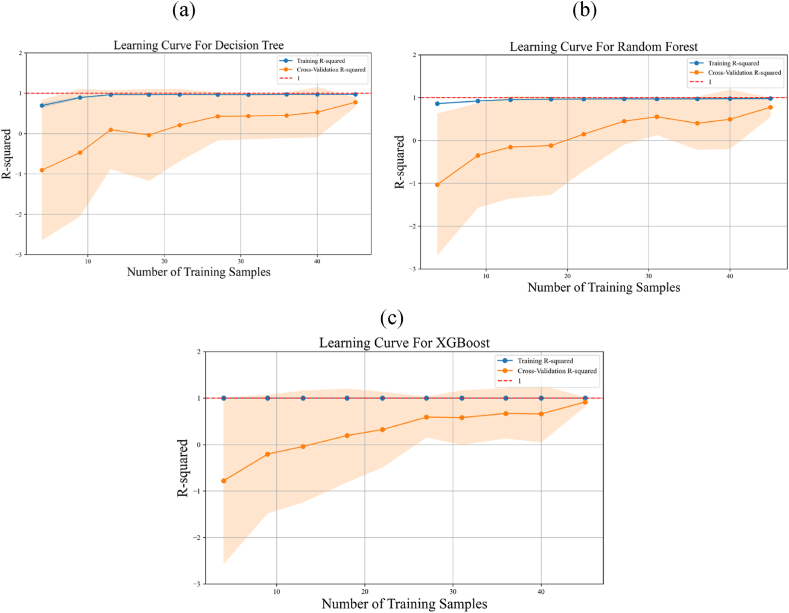
Learning curves for different ML methods based on training CFD dataset: (a) DT, (b) RF, and (c) XGBoost.

Finally, in Fig. 18, the learning curves for DT, RF, and XGBoost are presented. This figure illustrates low variance in training accuracy and testing accuracy, suggesting the absence of overfitting in these models [32]. Furthermore, after adding 40 samples for training the algorithms, the *R*^*2*^ value converges to 1. This indicates that the 54 different samples used in this study were appropriate for capturing the underlying patterns in the data.

### 3.4 Machine learning interpretation modeling

**Fig. 19:**
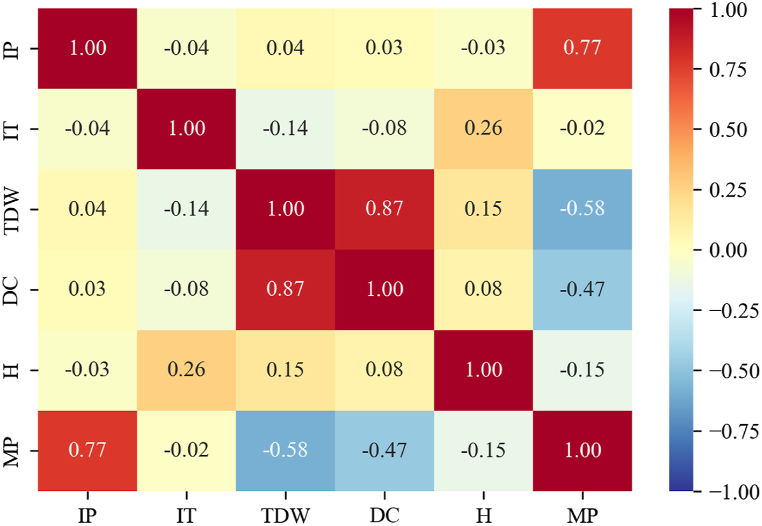
The pearson correlation matrix for the CDF dataset.

Fig. 19 illustrates the correlation matrix for the variables and output of the CFD dataset. This figure shows a strong and positive correlation between IP and MP. Additionally, it illustrates that other variables exhibit a negative correlation with MP.

To discuss the main reasons for using SHAP values as an interpreter of a complex algorithm like XGBoost in this paper, as compared to linear regression modeling and linear correlation or a simple sensitivity analysis, let's begin with the distinction between TDW and DC. These two features are different, with one being categorical and the other continuous. Both were identified as significant features. However, methods like SHAP values can demonstrate the effect of each specific feature without considering other features, based on cooperative game theory. Secondly, sensitivity analysis methods such as Pearson correlation cannot discern the impact of each feature in each specified sample. SHAP values, on the other hand, provide a more granular understanding of the contribution of each feature in the context of individual data points. Thirdly, a simple sensitivity analysis was unable to uncover the impact of changing nonlinear relations, such as the effect of DC on MP. SHAP values, being model-agnostic and capable of handling complex interactions, are better suited to capture and interpret these intricate nonlinear relationships within the data. In summary, SHAP values offer a more nuanced and comprehensive approach to interpreting the results of complex algorithms like XGBoost, especially when compared to linear regression modeling, linear correlation, or simplistic sensitivity analyses. Their ability to address the specifics of individual features and capture nonlinear relationships makes them a valuable tool for gaining insights into the workings of the model.

**Fig. 20:**
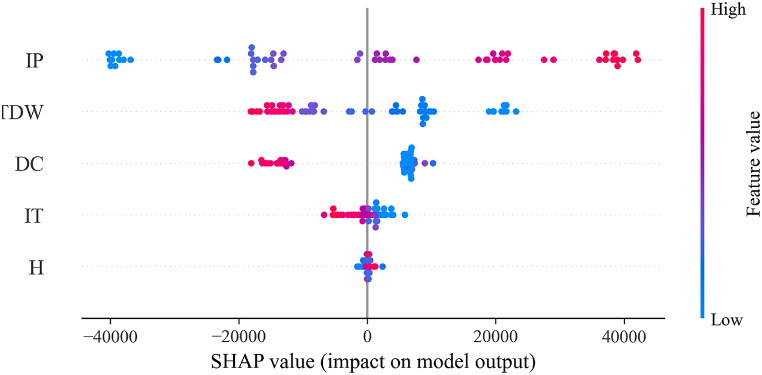
Beeswarm plot representing shap values.

**Fig. 21:**
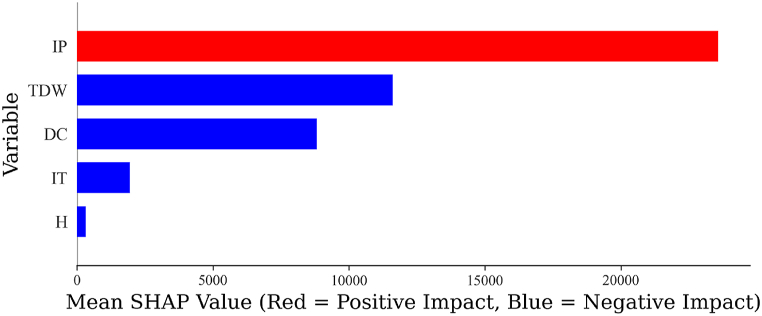
Average absolute SHAP values across various Input variables.

Therefore, Fig. 20 shows the Beeswarm plot of ML modeling with XGBoost. Beeswarm is a one-dimensional plot used for the interpretation of ML models; this method is employed in many research studies [[15], [16], [17],^28^,^34^]. This figure illustrates that IP had the most influence on the prediction, and the color of this variable indicates a positive impact on IP. Moreover, TDW had a lower influence compared to IP in predicting MP, and the color of this variable shows that TDW had a negative impact on the prediction of MP. DC holds the third position among the features affecting MP, and its color signifies a negative influence on the estimation of MP. The figure also indicates that H and IT are not significant variables in the MP of the internal arc of switchgear. Moreover, Fig. 21 depicts the mean absolute SHAP value for each specified variable among all the samples. This figure illustrates that, based on Equation (14), the fundamental constant value of the estimated MP undergoes an average change through the collection and subtraction of the values represented in this figure across these 54 samples. It also illustrates that the correlation matrix signs are aligned with SHAP values, demonstrating the relationship between variables and the target. Therefore, switchgear manufacturers should focus on increasing the area of ducts, separating the ducts, and changing the position of the ducts, compared to increasing the height of the switchgear. Moreover, adjusting the IP can help to reduce the pressure rise caused by internal arcs, while adjusting IT is not necessary. To discuss the results of the sensitivity analysis in this work in comparison with other research, Uzelac et al. [10] mentioned that in their study, based on the basic model, IP had a higher impact than IT in predicting MP. Moreover, duct area emerged as a significant factor affecting pressure elevation in both basic modeling and CFD modeling [1,10], and the area of the ducts is a function of TDW. Therefore, according to other research, TDW is also considered an important factor, aligning with this study.

**Fig. 22:**
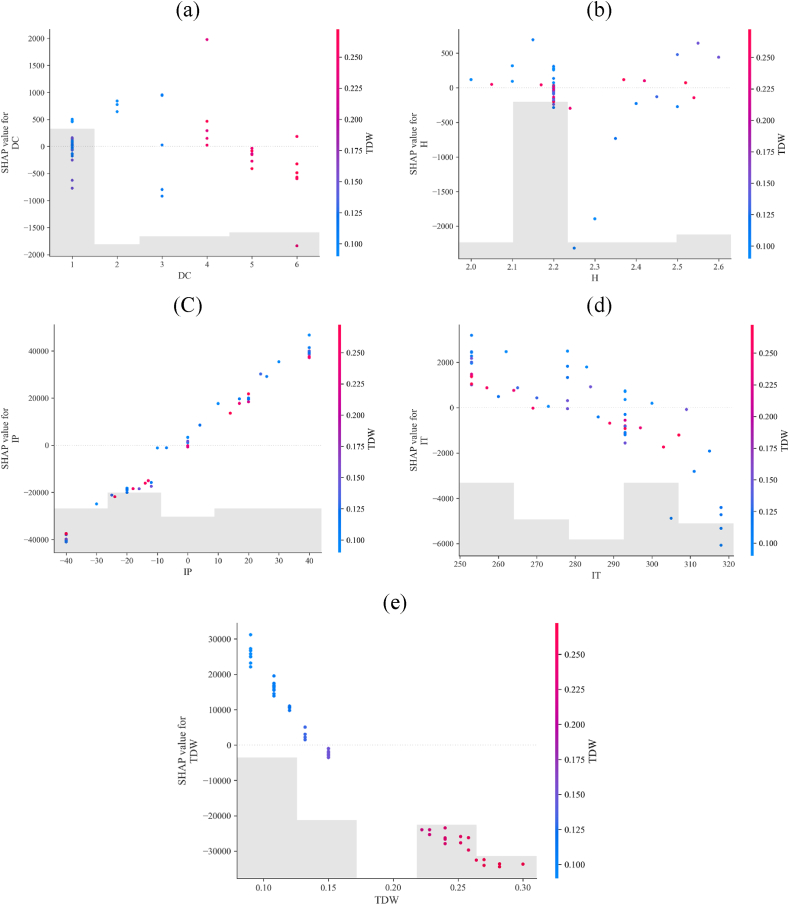
Histogram and scatter plot for variables in predicting MP: a) DC b) H c) IP d) IT e) TDW.

Fig. 22 illustrates scatter SHAP plots for each specified variable, and the color of each data point represents its TDW value. This visualization highlights that TDW, IP, and IT exhibit an approximately linear relationship between their SHAP values and actual values. Consequently, an increase in IP leads to an increase in MP, an increase in IT results in a decrease in MP, and an increase in TDW correlates with a decrease in MP. However, in DC and H, the results vary. Based on Fig. 22(a) and (e), the optimal value for DC, according to TDW, is when DC = 6 because this condition had the most negative SHAP compared to the others, and it decreased the MP as the objective. Moreover, there is not a clear conclusion or pattern for changing H and its impact on MP. It is worth mentioning that the low SHAP values for parameter H indicate minimal impact on the MP of the internal arc.

To discuss the pattern of changing SHAP values in Fig. 22 with other works, Chelgani et al. [17] illustrate a linear pattern for categorical features. However, in the present work, DC in Fig. 22 (a) exhibits non-linear behavior by altering its value, indicating the complexity of interpreting this feature. Furthermore, in the case of IT, IP, and TDW, the pattern is linear, as seen in earlier studies, including continuous features [15,17,28].

**Fig. 23:**
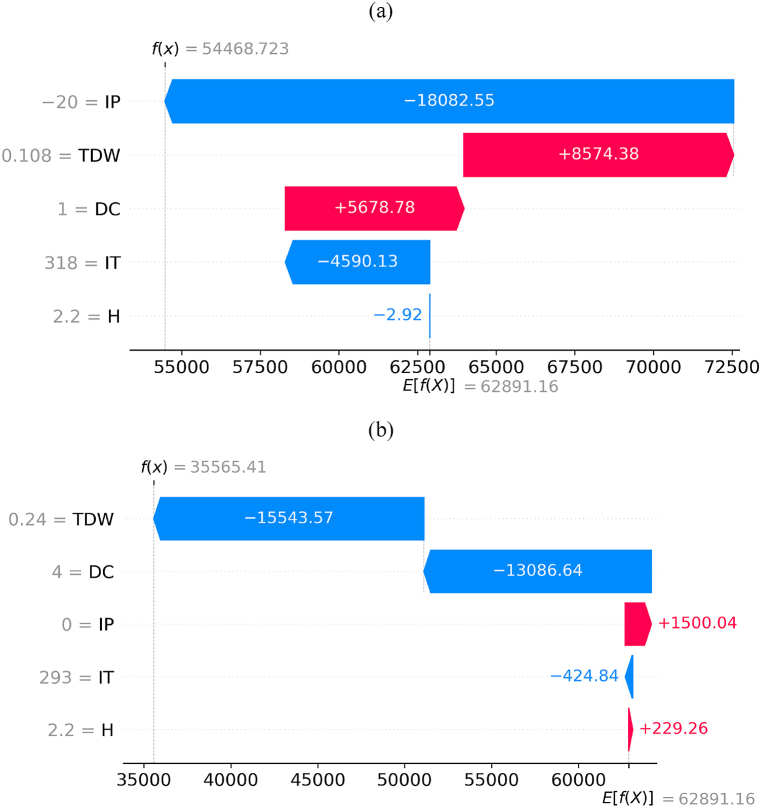
Waterfall plot for two different samples: (a) sample number 23, (b) sample number 47.

Fig. 23(a) and (b) illustrate how SHAP values, based on cooperative game theory, sum to the estimated baseline of MP, resulting in the estimated actual value of MP. Moreover, the baseline of MP (denoted as $\hat{y}$ in Equation (14)) is illustrated in these figures as E[f(X)] with a constant value of 62,891.16. Furthermore, the term f(x) represents the estimated actual value of MP (denoted as ${\hat{y}}_{0}$ in Equation (14)). To provide a more detailed explanation of Fig. 21(a) and (b), the estimated MP is demonstrated for sample number 23 inputs from the CFD dataset in Fig. 23(a) based on SHAP values. Additionally, Fig. 23(b) depicts the estimated value of MP for sample number 47 inputs based on SHAP values. These examples illustrate the SHAP values mechanism based on cooperative game theory to clarify the process of interpreting changes in feature values among the samples. Moreover, as an example based on Fig. 23(a), in sample number 23, IP, TDW, DC, IT, and H had the most impact on the estimation of MP based on the constant value of MP, respectively. In contrast, for sample number 47, TDW, DC, IP, IT, and H had the most impact on MP based on the constant value of MP, respectively.

### 3.5 Guidance for using AI in switchgear industry for optimization tasks

Nowadays, MV switchgear must comply with the International Electrotechnical Commission (IEC) standard, specifically IEC 62271-200. This IEC standard comprises several sections outlining routine tests and type test requirements, including internal arc type tests, short-time withstand current and peak withstand current type tests, temperature rise type tests, and more. Over the past few decades, numerous academics and technical teams have explored the design characteristics of switchgear based on specific requirements. For instance, studies have investigated the impact of switchgear design parameters on electrodynamic stress during short-time withstand current and peak withstand current tests [35,36], the influence of design parameters on temperature rises in continuous current tests [37,38], and the effects on pressure rises during internal arc tests, as reported in the present paper. These design parameters give rise to varying outcomes in the required tests and operations.

**Fig. 24:**
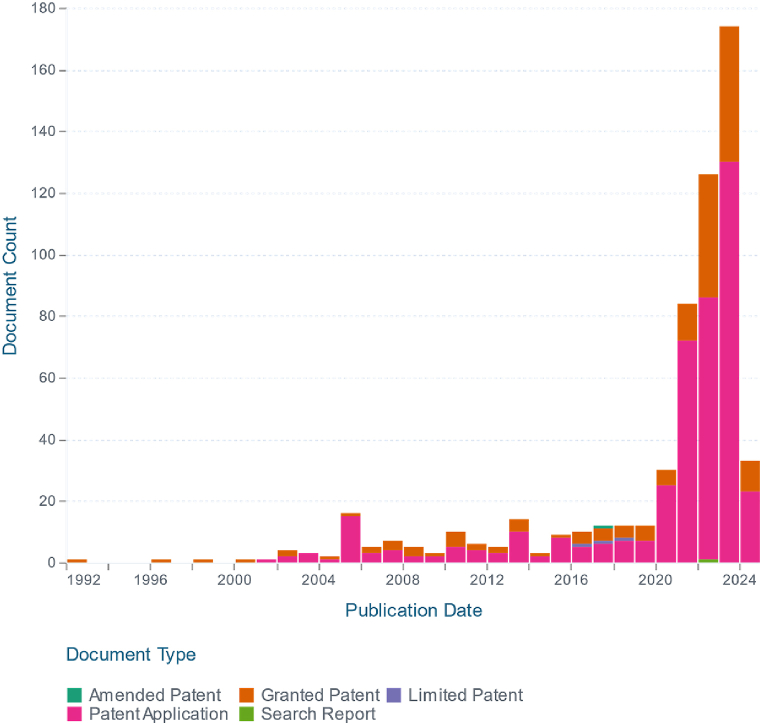
The trend in patent activity (Lens patent search: Artificial Intelligence in Switchgears).

From another perspective, the evolution of AI for optimization tasks [12] and the interpretation of complex models [39,40] has transformed the landscape across all fields of engineering science. The switchgear manufacturing industry is no exception and aligns with this trend. In Fig. 24, the phrase 'Artificial Intelligence in Switchgears' was searched on www.lens.org, and the figure illustrates the number of patent reports per year.

As mentioned in the present paper, this work aims to provide an AI interpretation of a multivariable design problem related to the maximum pressure (MP) during internal arcs in an MV switchgear. The main limitation of this work is its dependency on a specified MV geometry filled with air, which cannot be generalized to switchgear manufacturing with various product types. Furthermore, the primary design objective is to reduce the maximum pressure internally, and it is not a universal solution for all design requirements in switchgear. Therefore, the following statements will explain how to optimize switchgear based on specific geometry for each manufacturer, step by step:

• First of all, according to IEC standards, the objectives of the design parameters need to be specific. In the present work, the objective of the problem was to reduce the maximum pressure (MP) during internal arcs based on IEC 62271-200. However, these objectives may vary with other type test requirements.
• After determining the optimization objective, it is crucial to specify the design parameters and limitations. For instance, the width of air-insulated MV switchgear, particularly about their circuit breakers, is often challenging for manufacturers to alter comfortably. This paper analyzed the influence of four unique design elements and two installation parameters on the MP in internal arcs.
• The subsequent step involves finding a method to address the problem under various parameters. For instance, the finite element method and analytical approaches have proven effective in determining the mechanical stress caused by short-circuit currents in Short-time withstand current and peak withstand current tests [35,36]. In the present work, CFD modeling was employed to address this problem. While experimental modeling of switchgear tests remains the most reliable method, it is often deemed inappropriate for optimization tasks in the switchgear industry due to high associated costs.
• Following the selection of the problem solver, the problem must be solved utilizing a variety of design parameters. The present study investigated MP in 54 different runs of the CFD model with various input factors.
• If researchers can create a relationship between design parameters and the problem's objective, AI may not be necessary. Nevertheless, the switchgear design parameters exhibit interactions, and the presence of a high quantity of design variables serves as motivation for employing AI.
• The investigation of the problem's objective is crucial. If the problem entails more than one requirement, it is recommended to address multi-target regression problems using techniques such as ANN for modeling, as suggested by Refs. [11,12]. Conversely, if the problem's objective is focused on a single requirement, an algorithm like XGBoost could be beneficial for modeling a dataset with both categorical and continuous features. In the current study, the algorithm target for XGBoost is MP, and the objective of the problem is to reduce MP in internal arc conditions.
• Finally, the following approaches might work well for solving AI modeling interpretation and optimization. Using genetic algorithms in multi-target regression was recommended, as suggested by Ref. [11]. Conversely, for single-target regression problems, it is advised to utilize SHAP values for interpretation and optimization tasks, as recommended by Refs. [15,16]. Moreover, in the present study, SHAP values have been employed to interpret the effect of the internal arc parameters of MV switchgear on MP. The documentation on SHAP values (accessible at shap. readthedocs.io) greatly aids in understanding the AI interpretation mechanism, particularly for the specific design of MV switchgear based on the requirements of IEC 62271-200.

For future research, it is recommended to employ multi-target regression models using an ANN-based algorithm to predict internal pressure and temperature in switchgear. Propose optimizing the switchgear through a genetic algorithm with dual objectives focused on reducing pressure and temperature rise during internal arcs, potentially enhancing the current study's outcomes. Additionally, it suggests valuable future work involving the integration of IEC 62271-200 type test requirements as targets for simulation, modeling, and optimization of switchgear. As an example, investigate the impact of conductor thickness as a design parameter in switchgear, targeting mechanical stress in short-time withstand current and peak withstand current type tests, as well as temperature rise in continuous current tests.

The main limitation of the present work lies in reporting results on a specific geometry, which cannot be generalized to the switchgear industry with different products. However, guidance for each specified geometry has been reported in the present paper. Furthermore, in contrast to ANN-based algorithms, XGBoost, without using feature engineering and preprocessing approaches, typically requires training with a larger dataset. Nevertheless, XGBoost demonstrates compatibility with SHAP values compared to other methods.

## 4 Conclusions

This research centered on utilizing diverse machine learning (ML) methods to predict maximum pressure (MP) arising from an inadvertent fault in switchgear known as the 'Internal arc', using a generated computational fluid dynamics (CFD) dataset. Moreover, the predictions were conducted under various design and environmental adjustable parameters, and the influence of these parameters in predicting MP was determined based on Shapley additive explanation (SHAP) values. The subsequent results derived from this work are as follows:

• Comparing the Multiphysics CFD model, designed for a specified geometry and condition, with the basic model, the experimental test showed high accuracy. Furthermore, the models exhibited a satisfactory fit, as evidenced by the pressure rise plots, which depicted a maximum pressure (MP) of over 400,000 Pa within 1 s.
• The comparison of the experimental pressure rise in several MV switchgears reveals substantial variation in the MP values, which can be attributable to variations in their geometry and test conditions. Employing ML to interpret this phenomenon based on CFD simulations is motivated by this variation.
• The generated CFD dataset, based on the real switchgear geometry and conditions, contains 54 different samples representing distinct simulations. Each sample corresponds to a unique simulation with gage MP as the target. Additionally, the gage MP varies across all samples, ranging from 17835.45 Pa to 144423 Pa.
• The extreme gradient boosting (XGBoost) model demonstrated superior performance compared to other models, achieving a mean determination coefficient (*R*^*2*^) of 94.49 % for testing sets, a root means square error (RMSE) of 6625.25 for testing sets, and a scatter band (SB) of ±1.1 for all 54 training data points.
• The SHAP values revealed that initial pressure (IP), total duct width (TDW), ducts condition (DC), initial temperature (IT), and height (H) had the most significant to least significant effects on estimating maximum pressure (MP).
• SHAP values effectively addressed DC and TDW interference, unlike the correlation matrix and other simple methods that lacked specific solutions for this problem.

## Data availability

The raw CFD data for the trained machine learning models is presented in the Results and Discussion section. Furthermore, the Python codes for the ML models are available on request from the corresponding author.

## CRediT authorship contribution statement

**Mahmood Matin:** Writing – original draft, Methodology, Investigation, Formal analysis, Data curation. **Amir Dehghanian:** Writing – review & editing, Investigation, Formal analysis, Conceptualization. **Mohammad Dastranj:** Validation, Software, Investigation. **Hossein Darijani:** Writing – review & editing, Supervision.

## Declaration of competing interest

The authors declare the following financial interests/personal relationships which may be considered as potential competing interests Mahmood Matin, Amir Dehghanian, Mohammad Dastranj, Hossein Darijani reports financial support was provided by Kerman Tablo Co. If there are other authors, they declare that they have no known competing financial interests or personal relationships that could have appeared to influence the work reported in this paper.
